# Acute Focal Bacterial Nephritis in a Patient with Solitary Kidney: Case Report

**DOI:** 10.5811/cpcem.1545

**Published:** 2023-07-18

**Authors:** Adnane Guella, Rabab Zaka Muhammad, Mahdi Aljallabi, Abeer Mursi, Mohamed Elmahi

**Affiliations:** * University Hospital, Department of Internal Medicine, Sharjah, United Arab Emirates; † University Hospital, Department of Emergency Medicine, Sharjah, United Arab Emirates

**Keywords:** acute focal bacterial nephritis, acute lobar nephronia, case report

## Abstract

**Introduction:**

Acute focal bacterial nephritis is an underdiagnosed condition. It clinically resembles acute pyelonephritis. If unrecognized and undertreated, it may progress into complications (kidney abscess and scars). Contrast-enhanced computed tomography (CT) reveals specific images of the disease and is considered the gold standard to make the diagnosis.

**Case Report:**

A 63-year-old male patient with solitary kidney presented with symptoms compatible with acute pyelonephritis. Kidney ultrasound was not conclusive. Because of persisting high-grade fever not resolving after 48 hours of antibiotics, a contrast-enhanced CT was then performed, and the diagnosis of acute focal bacterial nephritis was made. A repeat CT after three weeks of intravenous (IV) antibiotics showed marked improvement of the intrarenal lesions, and a fourth week of IV antibiotics was dispensed.

**Conclusion:**

Diagnosing acute focal bacterial nephritis is important (particularly in a patient with solitary kidney). This will dictate the therapy duration. Unlike acute pyelonephritis, acute focal bacterial nephritis requires at least three weeks duration of antibiotics to avoid progress into further complications.

## INTRODUCTION

Acute focal bacterial nephritis (AFBN) was first described by Rosenberg et al. in 1979.^1^ It is defined as focal areas of non-liquefactive necrosis in the renal cortex that can involve one (focal) or more lobes (multifocal).^2^,^3^ Acute focal bacterial nephritis is considered to be a complicated form of acute pyelonephritis (APN) and lies on the spectrum between APN and renal abscess. It remains underdiagnosed in both adults and children. The main reason for underdiagnosis is the clinical similarity between APN and AFBN, and failure to order imaging. Contrast-enhanced computed tomography (CT) provides the most sensitive and specific images of AFBN, i.e., poorly enhancing, wedge-shaped lesion in the kidney. Ultrasonography can be helpful if nephromegaly or a focal mass are detected. Acute focal bacterial nephritis usually affects one kidney and, rarely, both kidneys. We report here a case in which AFBN is described in a patient with solitary kidney successfully managed by a long course of antibiotics.

## CASE REPORT

A 63-year-old male diabetic patient presented to the emergency department (ED) on two consecutive episodes. In the first presentation, he reported chills and dysuria for a few days but was not febrile. He reported slight right flank pain. He had been seen in a private clinic a few days before this presentation. At that time, he was febrile and received antibiotics. The most important element in his medical history was a left nephrectomy done in 2007 following a complicated course of kidney stone and infection. Therefore, he was classified as a mild case of APN and was discharged home on oral antibiotics for seven days.

Three days later, he again presented to the ED. This time, he had high-grade fever (40.1° Celsius) and severe right flank pain. There was no frank hematuria, nausea, or vomiting. On examination he had severe tenderness of the right costovertebral angle. His laboratory results showed leukocytosis (20,000/microliter (μL) (reference range: 4,000–10,000/μL) with markedly elevated C-reactive protein (196 milligrams (mg)/L [<10 mg/L]) and procalcitonin (6 nanograms (ng)/mL [≤ 0.15 ng/mL]). Kidney ultrasound confirmed the left nephrectomy with compensatory hypertrophic right kidney and no gross abnormalities apart from a rim of perinephric fluid. The urinary bladder was well distended with normal wall thickness. There was no evidence of mass, sludge, or stone. The prostate was of normal volume.

Blood and urine cultures were taken in the ED, and the patient was admitted as a case of APN and started empirically on meropenem at a dose of 1 gram three times daily as his kidney function was normal (creatinine was 105 micromoles [μmol]/L; reference range: 62–115 μmol/L). After 48 hours of antibiotics, the patient remained febrile with no improvement of the costovertebral tenderness. At this time, we received the results of the urine and blood cultures, which revealed an extended spectrum beta-lactamase (ESBL) *Escherichia coli* sensitive to meropenem. All these elements prompted a request for contrast-enhanced CT, which showed right renal ill-defined cortical hypodense areas with poor enhancement compatible with the diagnosis of multifocal bacterial nephritis (Image).

The patient’s condition improved after another 48 hours of meropenem. We then discharged him on ertapenem since its once daily administration is more convenient for outpatient treatment than the three daily doses of meropenem. A repeat contrast-enhanced CT after a three-week course of antibiotics revealed a remarkable improvement of the kidney lesions (Image). We decided to prescribe an additional week of ertapenem considering his severe condition and solitary kidney. After that, the patient was followed up monthly in the outpatient clinic and remained completely asymptomatic after six months of follow-up with normal kidney function.

**CPC-EM Capsule**

What do we already know about this clinical entity?

*Acute focal bacterial nephritis (AFBN) is underdiagnosed in adults as it resembles clinically acute pyelonephritis (APN).*

What makes this presentation of disease reportable?

We report a case of AFBN in a solitary kidney that if not diagnosed would have exposed the patient to serious complications

What is the major learning point?

*Diagnosing AFBN is easily made by contrast-enhanced computed tomography. A minimum three-week course of antibiotics is needed to clear the renal lesions.*

How might this improve emergency medicine practice?

*This case may help raise awareness and lower the threshold of suspicion for AFBN, particularly since APN is a common presentation in the emergency department.*

## DISCUSSION

Most cases of acute pyelonephritis are first seen in the ED. As AFBN shares with APN similar clinical presentation, it is seldom considered. Therefore, the patients do not proceed for imaging and are typically treated as outpatients for a short duration. Imaging is ordered only when nonspecific symptoms dominate the presentation, including nausea, severe vomiting, and abdominal guarding, mimicking other clinical conditions.^4^,^5^ Therefore, the diagnosis of AFBN may be delayed or even not considered. This is clearly shown in the study of Campos-Franco et al. The same expert radiologist retrospectively reviewed the images of 377 patients admitted at one hospital with APN over a five-year period. The diagnosis of AFBN was missed in 57 cases (prevalence of 15.1%) based on the ultrasound findings of renal focal mass(es) of decreased or, less frequently, increased echogenicity and decreased vascularity on Doppler or when contrast-enhanced CT revealed one or multiple wedge-shaped areas of decreased kidney density.^6^

Kidney ultrasound is the imaging of choice in children for its non-invasive nature, lack of radiation, and ability to detect congenital urological abnormalities. However, nephromegaly or the presence of an ill-defined focal mass may be missing. Saito et al reviewed AFBN cases in children seen in their facility from 2008–2011. They found that seven of 11 children had false negative renal ultrasound. The diagnosis of AFBN was made after contrast-enhanced CT, and the authors deemed CT to be indispensable for the diagnosis.^7^ In adults as well, ultrasonic anomalies may be lacking while contrast-enhanced CT findings are characteristic.^8^

In a recent systematic review reporting data from 138 cases, kidney ultrasound was performed in addition to CT and/or magnetic resonance imaging (MRI) in 41% and had a sensitivity of 91%. The diagnosis was confirmed solely by ultrasound in 20% of the cases, and 52% had their diagnosis confirmed by contrast-enhanced CT and/or MRI.^5^ Jiao et al. in their retrospective analysis of the data of 238 adult patients diagnosed with AFBN by contrast-enhanced CT, ultrasound identified nephromegaly in only 52 patients (21.85%) and a hypoechoic focal mass in two patients (0.84%), indicating that the sensitivity of ultrasound for AFBN diagnosis was probably not satisfactory.^9^ In our case, which to our knowledge is the first report of AFBN in a patient with solitary kidney, ultrasound did not show any focal mass, and the diagnosis was made by contrast-enhanced CT.

Our case highlights some aspects that should trigger the ordering of a contrast-enhanced CT, which is the gold standard for diagnosing AFBN. Many authors have tried to find potential markers or symptoms that would orient toward AFBN. Among these symptoms, fever present in 98% of the cases was found to be of higher grade (above 39° Celsius) and to persist for a longer period in AFBN than in APN.^6^,^9^ In our patient, fever was not present during the first presentation, which made AFBN an unlikely diagnosis. However, the patient had fever prior to this presentation when he visited a private clinic, and he received antibiotics for a few days. This may explain the absence of fever and the confusion in reaching a proper diagnosis. Symptoms during the second presentation were more serious and prompted further investigations.

In the management of AFBN, the duration of antibiotics therapy is important to consider. A treatment period for two weeks or less, as in APN, would be insufficient and may lead to further complications, particularly abscess formation and fibrosis.^10^,^11^ In pediatrics, it was clearly shown that a minimum of three weeks is mandatory for successful treatment of AFBN.^12^,^13^ Cheng et al observed 17% treatment failure with two weeks therapy duration vs 0% treatment failure with three weeks of antibiotic therapy.^13^ In adults, the optimal treatment period is not known, but in general the three- to four-week duration as in pediatrics is followed.^9^ We documented by CT the clearance of the focal lesions in the kidney after at least a three-week period of therapy.^14^ In our current case with solitary kidney, important multifocal lesions, and positive culture of ESBL *E. coli*, we opted for a longer treatment period with intravenous ertapenem for four weeks. We believe that this case should alert emergency physicians to have a lower threshold for AFBN suspicion, as the diagnosis of AFBN indicates the need for a longer duration of antibiotics.

## CONCLUSION

This case illustrates the usefulness of contrast-enhanced CT in diagnosing acute focal bacterial nephritis, particularly in patients with solitary kidney. Indeed, a longer treatment duration than in acute pyelonephritis should be considered to avoid further complications. Our observation confirms the recommendation that at least three weeks of antibiotics are required to clear the intrarenal infection process.

## Figures and Tables

**Image:**
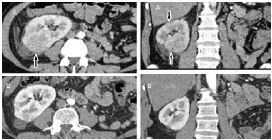
Contrast-enhanced computed tomography showing in “A” a wedge-shaped lesion with decreased enhancement (arrows), and in “B” marked improvement after three weeks of antibiotics.
